# Supplementary material for: Viruses affect picocyanobacterial abundance and biogeography in the North Pacific Ocean
Source: Nat Microbiol. 2022 Apr 1;7(4):570–80. doi: 10.1038/s41564-022-01088-x (PMC8975747; doi:10.1038/s41564-022-01088-x)
Supplement: Supplementary file 1 — Supplementary materials, discussion, Tables 1–4, Figs. 1–4 and refs. 90–108. [file 41564_2022_1088_MOESM1_ESM.pdf]

nature portfolio

https://doi.org/10.1038/s41564-022-01088-x

Supplementary information

Viruses affect picocyanobacterial abundance and biogeography in the North Pacific Ocean

In the format provided by the authors and unedited

Supplementary Information for

Viruses affect picocyanobacterial abundance and biogeography in the North Pacific Ocean

Michael. C. G. Carlson[1], François Ribalet[2], Ilia Maidanik[1], Bryndan P. Durham[2†], Yotam Hulata[1], Sara Ferrón[3], Julia Weissenbach[1], Nitzan Shamir[1], Svetlana Goldin[1], Nava Baran[1], B. B. Cael[3‡], David M. Karl[3], Angelicque E. White[3], E. Virginia Armbrust[2], and Debbie Lindell[1]*

[1] Technion – Israel Institute of Technology, Faculty of Biology, Haifa, Israel 3200003
[2] University of Washington, School of Oceanography, Seattle, Washington, USA 98195
[3] University of Hawaii at Manoa, Department of Oceanography, Honolulu, Hawaii, USA 96822
†current address: Department of Biology, Genetics Institute, University of Florida, Gainesville, Florida, USA 32610
‡ current address: National Oceanography Centre, European Way, SO14 3ZH Southampton, UK

*Correspondence to: dlindell@technion.ac.il

Supplementary Materials:

Supplementary Discussion 1-4
Tables S1-S4 5-8
Figures S1-S4 9-12
References (90-108) 13

Supplementary Discussion

Controls on the biogeography of *Prochlorococcus*

Temperature is the widely accepted parameter that controls *Prochlorococcus* distributions[11-13,15,90] with nutrient limitation[4,16,34,91] and mixed layer depth[14,92] implicated as well. Temperature related distribution of *Prochlorococcus* is well known both for the North Pacific Ocean[30-33] and globally[11,12,14,15,70]. Extensive distribution data for *Prochlorococcus* from thousands of field observations from previous field campaigns, as well as our data for the 2015 and 2016 cruises, suggest that *Prochlorococcus* would have been expected to inhabit waters in the transition zone in 2017 at abundances near 10^5 cells ml^{-1} at temperatures in the range of 15-18 °C [11,12,14,15,30-33,90]. Here we use the terminology of a decline in *Prochlorococcus* in relation to their expected distribution at these temperatures and do not intend to imply a time scale. To account for the seasonal migration of the transition zone and to provide a geographic point of reference, we compare *Prochlorococcus* abundances on the different cruises to the location of the chlorophyll front.

Biotic and abiotic controls on *Synechococcus'* distribution

Synechococcus populations decreased at warmer temperatures in June 2017 compared to the 2015 and 2016 transects (Fig. 2b). However, the shift in the 2017 decline occurred with a ~2 °C difference relative to the previous years, whereas the shift observed for the 2017 *Prochlorococcus* decline had a ~5 °C difference. The northernmost decline in *Synechococcus* corresponded to the region where picoeukaryotes reached ~2×10^4 cells·ml^{-1} in all three cruises (Extended Data Fig. 2), suggesting that competition with picoeukaryotes may contribute to the *Synechococcus* decline and the spatial succession between these two phytoplankton groups. Although none of the environmental parameters measured correlated with the decline in *Synechococcus* in 2017, factors such as mixed layer depth or as of yet unknown abiotic conditions may also be important in the decline in *Synechococcus.* It is important to note that temperature is a less significant driver of the overall distribution range of the *Synechococcus* genus than for the *Prochlorococcus* genus[11,12,46,90], at least at the temperature ranges relevant for this region.

Single-virus and single-cell infection quantification of the dominant cyanophage lineages

The polony method uses highly degenerate primers and probes to capture diverse viruses from the major cyanophage lineages quantified in this study. These primers and probes were designed using both cyanophage isolates and environmental sequences and capture environmental virus genotypes across the diversity of the cyanophage lineages examined[37,38]. We previously verified that that these primers and probes match cyanophage sequences from

metagenomic datasets *in silico*[39]. Specifically, polony primers and probes were compared to the diversity of cyanophages in 44 metagenomic samples collected in the North Pacific Subtropical Gyre in July 2015[42] and found that 93% of cyanophage reads would be detected when allowing for 2 mismatches[39]. Previous studies have shown that primers with up to 2-4 mismatches can be tolerated in PCR assays without detrimental effects[93,94]. Additionally, we empirically tested the sensitivity of mismatches to probe hybridization and found that up to 3 mismatches can be tolerated in the polony assay. Notably, the relative cyanophage community composition in the metagenomes was similar to that found using single-virus quantification with the polony method.

Here, we further investigated the suitability of our primers and probes for capturing cyanophage diversity across the transects in the North Pacific Ocean traversed in this study. Viral metagenomes (n=68) were collected in parallel with polony samples across the 2016, 2017, and 2019 transects. We used a phylogenetic-based approach to classify assembled sequences (Supplementary datasets 2-7) that represent the dominant viruses in the water at the time of sampling, as described previously[39]. The polony assay was able to capture 99% (1233/1238), 93% (296/317), and 96% (368/385) of T4-like cyanophage sequences with ≤2 mismatches to the forward primer, reverse primer, and probe sequences, respectively. Similarly, the polony assay was able to capture 98% (391/397), 95% (160/167), and 98% (276/283) of T7-like cyanophage sequences with ≤2 mismatches to the forward primer, reverse primer, and probe sequences, respectively. These calculations are conservative estimates of the cyanophage diversity captured as they do not consider sequencing error that may underlie some of the observed sequence variability in the metagenomes. Thus, our primers and probes may actually detect more than 93% of the sequence variants. No assemblies were detected for TIM5-like cyanophages, likely due to their low abundance.

Our polony methods are aimed at quantifying the dominant cyanophage groups in the oceans. T4-like and T7-like cyanophages are the dominant cyanophages in metagenomic datasets from global expeditions such as the Tara Oceans Expedition[43] as well as specifically from surface waters in the North Pacific Subtropical Gyre[39,42]. Other phage lineages known to infect picocyanobacteria are expected to contribute little to cyanobacterial mortality. For example, the cyanosiphoviruses are a minor component of the cyanophage community (<10%) in the surface ocean[39,42] as are other non-T4-like or T7-like cyanophages[44,95-98] discovered over the last decade, including the TIM5-like cyanophages[44]. Furthermore, the handful of infected picocyanobacteria detected from the sequencing of hundreds of single cell genomes of *Prochlorococcus* and *Synechococcus* were infected by T4-like and T7-like cyanophages, including the cells collected across the transects on the 2016 cruise[41]. We cannot definitively rule out the contribution of these non-T4-like or T7-like cyanophage or yet to be discovered virus types as important contributors to mortality at this time using our targeted approaches. However, based on the above independent lines of evidence, these other cyanophages are likely to be rare relative to the T4-like and T7-like cyanophages.

Estimating infection and mortality from iPolony measurements
Infection values were calculated and mortality was estimated from instantaneous measurements of infection in the environment. This was done for each of the cyanophage lineages infecting a particular host genus (e.g. T4-like cyanophages that infect *Prochlorococcus*). Cyanophages in the environmental likely exhibit a range of latent periods. Since it is not possible to measure latent periods *in situ*, we used the latent periods determined from cyanophage infections carried out under laboratory conditions (n=24, Table S3)[39,78]. Average values were used as we expect them to reflect the diversity of latent periods of cyanophages in the environment.

The calculation of percent infection from iPolony data uses the relative proportion of the latent period, divided into three bins representing the periods prior to, during, and after phage genome replication which have different detection efficiencies due to differences in the copy number of phage genomes inside the cell[39]. Testing the sensitivity of this calculation to the variability in the length of time allocated to each bin changed infection values reported in this manuscript by an average of 0.0006% for T4-like cyanophages when bins were lengthened or shortened by one standard deviation. Thus, this variability appears to have a minimal impact on our percent infection values. The main source of uncertainty is derived from differential detection across the latent period, which results in a 2-fold maximal difference in values if infections in environmental populations were highly synchronized, either prior to or after phage genome replication[39]. These bounds of uncertainty in infection values are shown by the error bars in Extended Data Fig. 5 and Extended Data Fig. 6.

Estimating mortality depends on the number of infection cycles cyanophages are expected to complete in one day. The latent period averages used for the T4-like and T7-like cyanophages are closer to the longer end of the distribution suggesting our mortality estimates are likely to be conservative. If cyanophage communities were composed of a few dominant genotypes whose latent periods were significantly different from the average, this would affect our mortality estimates. Determining the suitability of these averages for our estimates awaits the ability of the field to estimate cyanophage latent periods in field settings.

Expected shifts in picocyanobacterial diversity along environmental gradients
Picocyanobacterial community composition is expected to undergo changes in the vicinity of the transition zone. While, the high light (HL) II *Prochlorococcus* ecotype dominates in the subtropics[12-15,30,33], the HLI ecotype is expected to become the most abundant *Prochlorococcus* ecotype due to higher growth rates at the temperature range of 16-18 °C found in the transition zone[12,15,30]. There may also be changes in *Prochlorococcus* diversity within an ecotype, such as the enrichment of *Prochlorococcus* HLI.2 ecotypes during summer months[30]. *Synechococcus* clades are likely to undergo similar reorganizations in their community structure in the transition zone. The oligotrophic specialist *Synechococcus* clades II and III are expected to be succeeded by the cold-water adapted clades I and/or IV at ~15 °C based on previous findings for the Pacific and Atlantic Oceans[33]. Additionally, the low iron adapted clade CRD1 has been previously observed to thrive in the North Pacific inter-gyre transition zone region[33].

Virus-mediated organic matter production

In the subtropical gyre, low levels of infection of *Prochlorococcus* contributed to an average of 9.0±6.7 μmol $C\cdot m^{-3}\cdot d^{-1}$, 1.2±0.94 μmol $N\cdot m^{-3}\cdot d^{-1}$ and 0.04±0.04 μmol $P\cdot m^{-3}\cdot d^{-1}$ released by viral lysis. Assuming a lysed cell is quickly converted into dissolved organic matter[59] and that a lysed picocyanobacterium has a C:N:P stoichiometry similar to that of growing cells (see Methods), viral lysis of picocyanobacteria would account for ~13%, ~4% and ~2% of the production of daily dissolved organic carbon (DOC), organic nitrogen, and organic phosphorus, respectively, based on previously measured organic matter production rates in the North Pacific Subtropical Gyre and other subtropical gyres[81-83]. As infection levels and relative contribution of *Synechococcus* increased in the transition zone, organic matter production was estimated, on average, to be up to 20-fold greater than the subtropics, reaching between 23-174 μmol $C\cdot m^{-3}\cdot d^{-1}$ (46±42 μmol $C\cdot m^{-3}\cdot d^{-1}$ average).

Table S1. Comparison between predictive models for cyanophage abundances

Predicted variable	Model	RMSE
Total cyanophages	*Prochlorococcus* × Temperature	0.94
	Synechococcus × Temperature	0.55
	Chlorophyll × Temperature	0.50
	Pro:Syn ratio × Temperature	0.40
	Total cyanobacteria × Temperature	1.2
T4-like cyanophages	*Prochlorococcus* × Temperature	1.4
	Synechococcus × Temperature	0.66
	Chlorophyll × Temperature	1.0
	Pro:Syn ratio × Temperature	0.79
	Total cyanobacteria × Temperature	1.6
T7-like cyanophages	*Prochlorococcus* × Temperature	1.9
	Synechococcus × Temperature	1.1
	Chlorophyll × Temperature	1.5
	Pro:Syn ratio × Temperature	1.2
	Total cyanobacteria × Temperature	2.2

Table S2. Primers and probes used in polony methods[37,38]

	Target phages	Target gene	Name	Sequence	Modification	Concentration (µM)	Thermal cycling conditions*
Primers	T7-like cyanophages	DPOL	534Rd	TGNWRYTCRTCRTGNAYRAA	5'-Acrydite	20	94 °C for 5 m, then 50 cycles of 94 °C for 45 s, 50 °C for 45 s, 72 °C for 2 m then 72 °C for 6 m
			341Fd-15-NNN	NNNCCNAAYYTNGSNCAR		15	
	T4-like cyanophages	g20	CPS1.2	ATHTTYTAYATHGAYGTNGG	5'-Acrydite	10	94 °C for 5 m, then 50 cycles of 94 °C for 45 s, 35-50 °C by 0.3 °C steps for 45 s, 72 °C for 2 m then 72 °C for 6 m
			CPS8.2	ARTAYTTNCCNRYRWANGG		10	
	TIM5-like cyanophages	DPOL	Dpol2_2F	YWYGCNTAYAAYGARATG	5'-Acrydite	20	94 °C for 5 m, then 50 cycles of 94 °C for 45 s, 50 °C for 45 s, 72 °C for 2 m then 72 °C for 6 m
			Dpol2R	CCANGCRTTNGCNWSNGG		10	
Probes	Clade A T7-like cyanophages	DPOL	405AF(d+3i)	TAYTGYYTIATITAYGGIGG	5'-Cy3	1.2	94 °C for 6 m, then 42 °C for 30 m
	Clade B T7-like cyanophages (w/o Tip42 subclade)		405BF(d+3i)	TAYGCITTYYTITAYGGIGC	5'-Cy5	0.45	
	Tip42 subclade T7-like cyanophages		405BF(d+3i)tip42	TAYTGITTYYTITAYGGIGG	5'-Cy5	0.15	
	T4-like cyanophages and some non-cyano T4-like environmental sequences	g20	g20_cyano_env	RTCRTAYTGDATRTGITC	5'-Cy5	0.6	94 °C for 6 m, then 42 °C for 30 m
	Some non-cyano T4-like environmental sequences		g20_env	GCRAARTRICCRTCYTK	5'-Cy3	1.2	
	TIM5-like cyanophages	DPOL	TIM5-C(d+2i)	GAYTGiATRCACCAiTTRTT	5'-Cy5	0.6	94 °C for 6 m, then 42 °C for 30 m
			TIM5-V(d+2i)	GAYTGRATiACCCAiTTRTT	5'-Cy5	0.6	

*Note that thermal cycling for iPolony reactions (infected cell assays) was initiated with 94 °C for 15 m then 25 °C for 15 m prior to the conditions listed above.

Table S3. Infection dynamics for cyanophages infecting marine picocyanobacteria

Virus group	Host	Latent period (h) (n)	Virus production (viruses $cell^{-1}$)* (n)	References
T4-like	*Synechococcus*	8.9 ± 4.5 (7)	42 ± 12 (3)	[22,66,99-103]
T4-like	*Prochlorococcus*	8.8 ± 1.1 (5)	12 ± 0 (2)	[22,103-106]
T7-like clade A	*Synechococcus*	3.3 ± 3.3 (5)	97 ± 15 (5)	[103], (Maidanik et al., *unpubl. data*)
T7-like clade A	*Prochlorococcus*	n/a[†]	n/a[†]	
T7-like clade B	*Synechococcus*	7.0 ± 2.3 (5)	55 ± 29 (5)	[103], (Maidanik et al., *unpubl. data*)
T7-like clade B	*Prochlorococcus*	8.6 ± 1.1 (5)	53 ± 18 (2)	[107,108], (Maidanik et al., *unpubl. data*)

* Virus production is measured by three different approaches, measuring either infective viruses, total particles, or free and packaged genomic DNA.

[†] There are no published latent periods and burst sizes of T7-like clade A cyanophages on *Prochlorococcus*. For use in mortality estimates, we assumed they had similar average latent periods to *Synechococcus* infecting T7-like clade A cyanophages. We also assumed that the average burst size was 1.9-fold higher than *Prochlorococcus*-infecting T7-like clade B cyanophage based on the fold difference observed between *Synechococcus* infecting T7-like clade A and B cyanophages.

Table S4. Model parameter values

Parameter	Total cyanophages	T7-like cyanophages	T4-like cyanophages
a_0	0.2	-10.3	3.5
a_1	0.88	1.5	0.61
a_2	-5.47	-9.98	-3.54
a_3	0.36	0.64	0.23

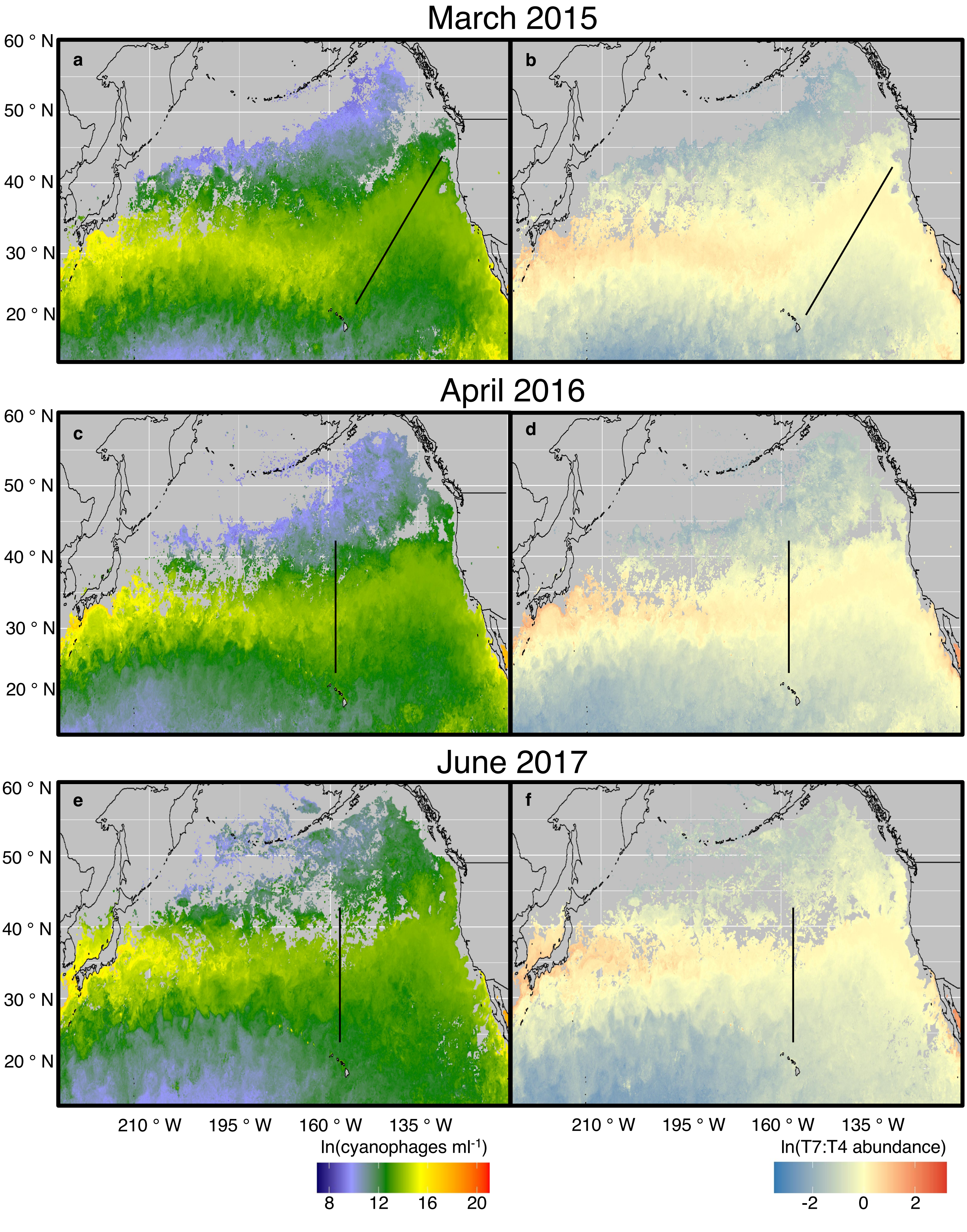

Fig. S1. Predicted cyanophage abundances in the North Pacific Ocean. The regression model was trained on samples collected along the 2015, 2016 and 2019 transects. (**a, b**) March 2015, (**c, d**) April 2016, and (**e, f**) April 2019. Total cyanophages (**a, c, e**) and the T7-like:T4-like cyanophage ratio (**b, d, f**). The hotspot peak corresponds to yellow regions in (**a, c, e**) and red regions in (**b, d, f**).

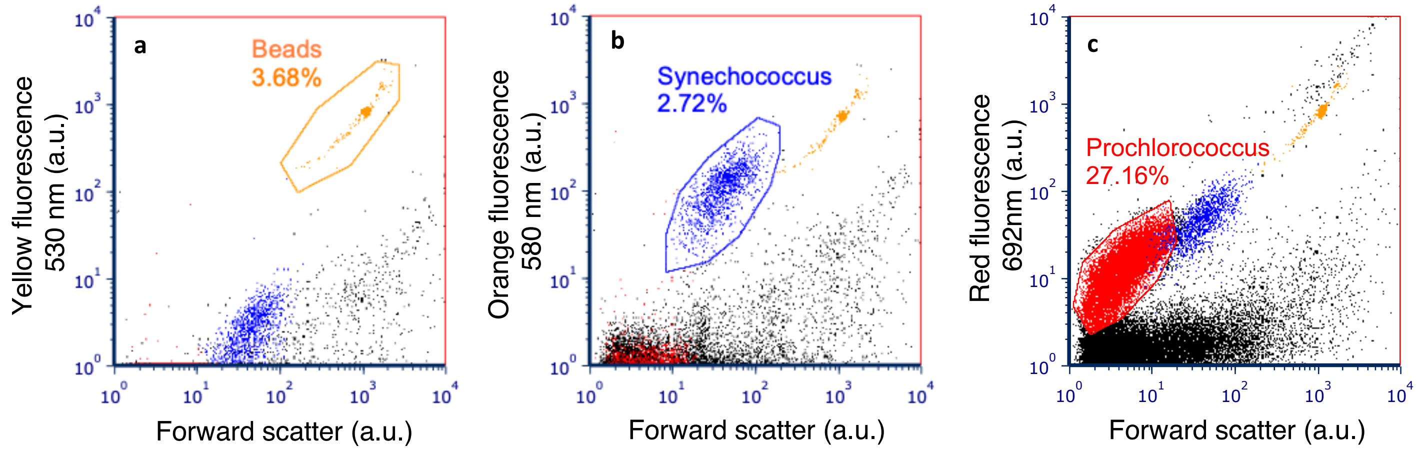

Fig. S2. Representative cytograms and gating of open ocean microbial communities. Picocyanobacteria are detected and gated based on size (forward scatter) and their natural autofluorescent pigments. Hierarchical gating was employed to first discriminate yellow-green beads added as an internal standard (a), then *Synechococcus* based on the orange fluorescence of phycoerythrin (b), and finally *Prochlorococcus* based on size, chlorophyll fluorescence and the lack of orange fluorescence (c). Optical properties (fluorescence and scatter) are measured in arbitrary units (a.u.).

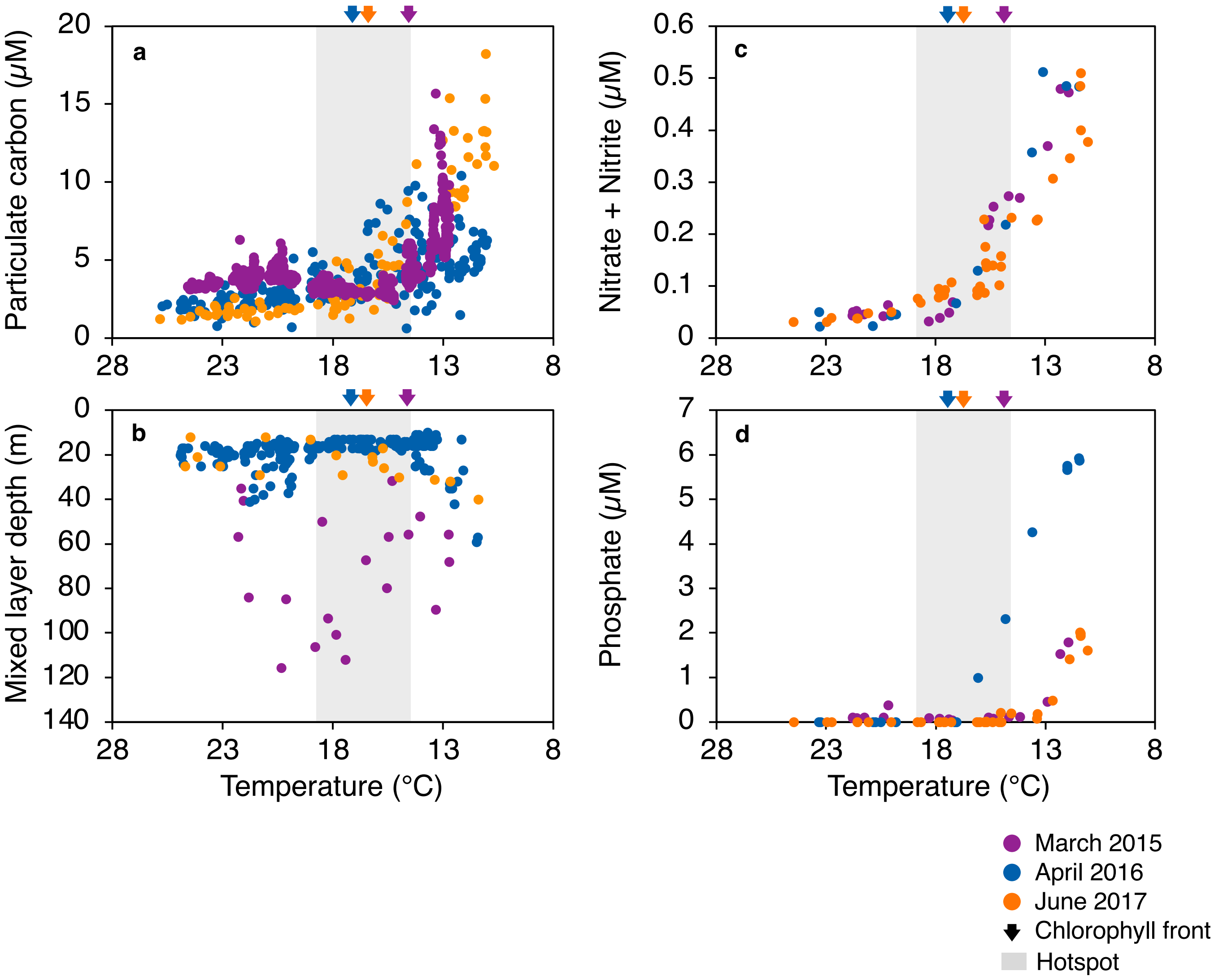

Fig. S3. Changes in physiochemical water column properties with temperature in the North Pacific Ocean. Particulate carbon (**a**), mixed layer depth (**b**), nitrate + nitrate concentrations (**c**), and phosphate concentrations (**d**) from the March 2015 (purple), April 2016 (blue), and June 2017 (orange) transects. Shaded regions indicate the hotspot and arrows indicate the chlorophyll front position.

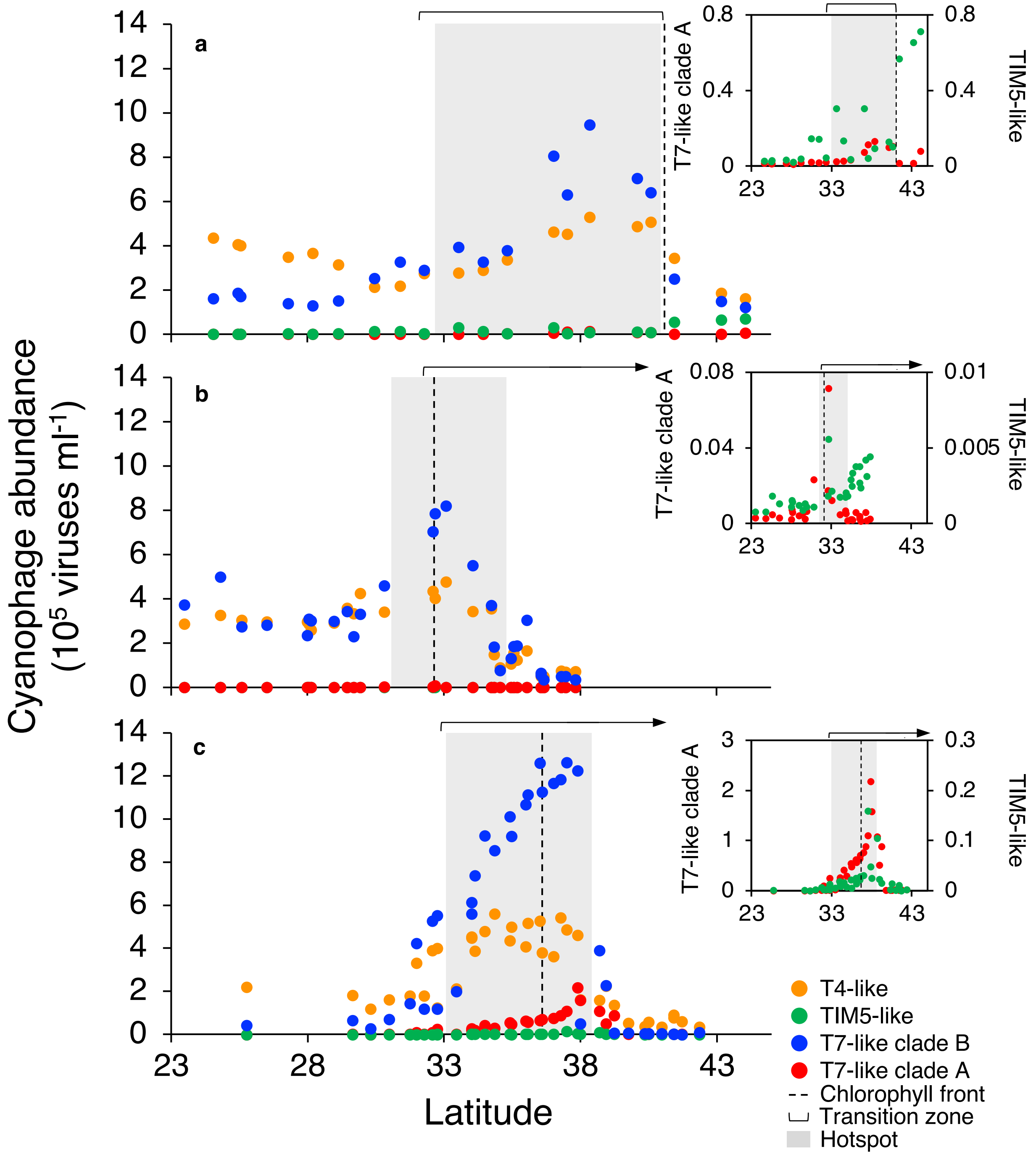

Fig. S4. Cyanophage community composition plotted against latitude across the North Pacific Gyres. March 2015 (**a**), April 2016 (**b**), and June 2017 (**c**) transects. Insets show T7-like clade A and TIM5-like cyanophage abundances on a zoomed scale. Note that all vertical axes, including insets, are plotted with units of 10^5 viruses·ml^{-1}. The dashed lines and shaded regions show the position of the chlorophyll front and the virus hotspot, respectively. See Extended Data Fig. 4 for confidence intervals and out-and-back reproducibility.

References

90 Zwirglmaier, K. *et al.* Global phylogeography of marine *Synechococcus* and *Prochlorococcus* reveals a distinct partitioning of lineages among oceanic biomes. *Environ. Microbiol.* **10**, 147-161, (2008).

91 Kent, A. G. *et al.* Parallel phylogeography of *Prochlorococcus* and *Synechococcus*. *ISME J.* **13**, 430-441, (2019).

92 Lindell, D. & Post, A. F. Ultraphytoplankton succession is triggered by deep winter mixing in the Gulf of Aqaba (Eilat), Red Sea. *Limnol. Oceanogr.* **40**, 1130-1141, (1995).

93 Stadhouders, R. *et al.* The effect of primer-template mismatches on the detection and quantification of nucleic acids using the 5' nuclease assay. *J. Mol. Diagn.* **12**, 109-117, (2010).

94 Christopherson, C., Sninsky, J. & Kwok, S. The effects of internal primer-template mismatches on RT-PCR: HIV-1 model studies. *Nucleic Acids Res.* **25**, 654-658, (1997).

95 Mizuno, C. M., Rodriguez-Valera, F., Kimes, N. E. & Ghai, R. Expanding the marine virosphere using metagenomics. *PLoS Genet.* **9**, e1003987, (2013).

96 Chenard, C., Chan, A. M., Vincent, W. F. & Suttle, C. A. Polar freshwater cyanophage S-EIV1 represents a new widespread evolutionary lineage of phages. *ISME J* **9**, 2046-2058, (2015).

97 Xu, Y. *et al.* Novel phage–host interactions and evolution as revealed by a cyanomyovirus isolated from an estuarine environment. *Environ. Microbiol.* **20**, 2974-2989, (2018).

98 Flores-Uribe, J. *et al.* A novel uncultured marine cyanophage lineage with lysogenic potential linked to a putative marine *Synechococcus* 'relic' prophage. *Environ. Microbiol. Rep.* **11**, 598-604, (2019).

99 Doron, S. *et al.* Transcriptome dynamics of a broad host-range cyanophage and its hosts. *ISME J* **10**, 1437-1455, (2016).

100 Fedida, A. & Lindell, D. Two *Synechococcus* genes, two different effects on cyanophage infection. *Viruses* **9**, (2017).

101 Bailey, S., Clokie, M. R., Millard, A. & Mann, N. H. Cyanophage infection and photoinhibition in marine cyanobacteria. *Res. Microbiol.* **155**, 720-725, (2004).

102 Puxty, R. J., Millard, A. D., Evans, D. J. & Scanlan, D. J. Viruses inhibit CO_2 fixation in the most abundant phototrophs on Earth. *Curr. Biol.* **26**, 1585-1589, (2016).

103 Wang, K. & Chen, F. Prevalence of highly host-specific cyanophages in the estuarine environment. *Environ. Microbiol.* **10**, 300-312, (2008).

104 Liu, R., Liu, Y., Chen, Y., Zhan, Y. & Zeng, Q. Cyanobacterial viruses exhibit diurnal rhythms during infection. *Proc. Natl. Acad. Sci. USA* **116**, 14077-14082, (2019).

105 Thompson, L. R., Zeng, Q. & Chisholm, S. W. Gene expression patterns during light and dark infection of *Prochlorococcus* by cyanophage. *PLoS ONE* **11**, e0165375, (2016).

106 Fridman, S. *et al.* A myovirus encoding both photosystem I and II proteins enhances cyclic electron flow in infected *Prochlorococcus* cells. *Nat. Microbiol.* **2**, 1350-1357, (2017).

107 Lindell, D., *et al.* Genome-wide expression dynamics of a marine virus and host reveal features of co-evolution. *Nature*, **449**, 83–86 (2007).

108 Frois-Moniz, K. *Host/virus interactions in the marine cyanobacterium Prochlorococcus*. PhD thesis, (Massachusetts Institute of Technology, 2014).
