# Supplementary material for: Viruses affect picocyanobacterial abundance and biogeography in the North Pacific Ocean
Source: Nat Microbiol. 2022 Apr 1;7(4):570–80. doi: 10.1038/s41564-022-01088-x (PMC8975747; doi:10.1038/s41564-022-01088-x)
Supplement: Supplementary file 2 — Reporting Summary [file 41564_2022_1088_MOESM2_ESM.pdf]

Corresponding author(s): Debbie Lindell
Last updated by author(s): Feb 2, 2022

Reporting Summary

Nature Portfolio wishes to improve the reproducibility of the work that we publish. This form provides structure for consistency and transparency in reporting. For further information on Nature Portfolio policies, see our Editorial Policies and the Editorial Policy Checklist.

Statistics

For all statistical analyses, confirm that the following items are present in the figure legend, table legend, main text, or Methods section.

n/a	Confirmed	
☐	☒	The exact sample size (*n*) for each experimental group/condition, given as a discrete number and unit of measurement
☐	☒	A statement on whether measurements were taken from distinct samples or whether the same sample was measured repeatedly
☐	☒	The statistical test(s) used AND whether they are one- or two-sided<br>*Only common tests should be described solely by name; describe more complex techniques in the Methods section.*
☒	☐	A description of all covariates tested
☒	☐	A description of any assumptions or corrections, such as tests of normality and adjustment for multiple comparisons
☐	☒	A full description of the statistical parameters including central tendency (e.g. means) or other basic estimates (e.g. regression coefficient) AND variation (e.g. standard deviation) or associated estimates of uncertainty (e.g. confidence intervals)
☐	☒	For null hypothesis testing, the test statistic (e.g. *F*, *t*, *r*) with confidence intervals, effect sizes, degrees of freedom and *P* value noted<br>*Give P values as exact values whenever suitable.*
☒	☐	For Bayesian analysis, information on the choice of priors and Markov chain Monte Carlo settings
☒	☐	For hierarchical and complex designs, identification of the appropriate level for tests and full reporting of outcomes
☐	☒	Estimates of effect sizes (e.g. Cohen's *d*, Pearson's *r*), indicating how they were calculated

Our web collection on statistics for biologists contains articles on many of the points above.

Software and code

Policy information about availability of computer code

Data collection	Continuous flow cytometery measurements were collected using SeaFlow software (Swalwell et al. 2011)<br>Discrete flow cytometry data were acquired using FACSDiva v8 or Spigot software
Data analysis	Continuous flow cytometry data were analyzed with the R package popcycle v1.1<br>Discrete flow cytometry data were analyzed using FACSDiva v8 or Spigot software<br>Polony data were analyzed using GenePix Pro v 5.0 and ImageJ v 1.0<br>Microscopy data were analyzed using Leica Application Suite X and ImageJ v 1.0<br>Satellite data were analyzed using SeaDAS v7.5.3 and cyanophage map data was analyzed using R packages 'stats' v 3.6.2, 'mapdata' v 2.3.0, and 'ggplot' v 5.5.5<br>Nutrient concentrations were analyzed using AQ software v 2.4.5<br>RStudio v 1.2.5019 and Python v 3.8.2 were used to analyze discrete data

For manuscripts utilizing custom algorithms or software that are central to the research but not yet described in published literature, software must be made available to editors and reviewers. We strongly encourage code deposition in a community repository (e.g. GitHub). See the Nature Portfolio guidelines for submitting code & software for further information.

Data

Policy information about availability of data

All manuscripts must include a data availability statement. This statement should provide the following information, where applicable:

- Accession codes, unique identifiers, or web links for publicly available datasets
- A description of any restrictions on data availability
- For clinical datasets or third party data, please ensure that the statement adheres to our policy

The data that support the findings of this study are available at https://simonscmap.com/catalog/cruises/ in the directories KM1502, KOK1606, MGL1704, KM1906. Additionally, discrete data presented here are provided in Supplementary dataset file 1.

Field-specific reporting

Please select the one below that is the best fit for your research. If you are not sure, read the appropriate sections before making your selection.

☐ Life sciences ☐ Behavioural & social sciences ☒ Ecological, evolutionary & environmental sciences

For a reference copy of the document with all sections, see nature.com/documents/nr-reporting-summary-flat.pdf

Ecological, evolutionary & environmental sciences study design

All studies must disclose on these points even when the disclosure is negative.

Study description

Latitudinal surveys of microbial and viral abundances and infection along 5 transects in 3 cruises between 2015-2017 in the North Pacific Ocean. Data from a fourth cruise in 2019 was used for validation of the multiple regression model built using data from the previous 3 cruises.

Research sample

Open ocean microbes and viruses and associated environmental conditions to investigate the effect of environmental conditions on cyanophage abundance and impact on picocyanobacteria. The samples represent the populations in the water masses they were collected in.

Sampling strategy

Sampling was performed at ~5m depth at high spatial resolution to assess the changes in microbial and viral dynamics along environmental gradients. Cruise tracks were designed to traverse the changing environment between the North Pacific Subtropical and Subpolar Gyres. Sampling schemes were designed such that the sample size collected in each regime would be statistically robust (n>=5) and occur at regularly spaced intervals.

Data collection

Samples were collected in the North Pacific Ocean from March 21 - March 29 2015 and April 10 - April 29 2019, from April 20 - May 4 2016, and from May 26th - June 13th 2017. Temperature and salinity were collected by shipboard sensors. Nutrients were collected into acid-cleaned high-density polyethylene bottles using a CTD rosette equipped with Niskin® bottles closed at 15 m depth. The samples were immediately frozen at -20 ºC until analysis in the laboratory. All samples used in virus analyses (VLPs, cyanophage abundances, and infection) were collected from each ship's flow-through seawater system located at ~5 m depth.

For analyses of viral abundance and infection levels, water was first filtered through a 20 µm mesh. Ten milliliter samples were collected for iPolony infected cell analysis, amended with glutaraldehyde (0.1% final concentration), incubated at 4 ºC in the dark for 15-30 minutes, snap frozen in liquid nitrogen, and stored at -80 ºC. Forty milliliter samples were filtered through a 0.2 µm syringe top filter to collect the virus-containing filtrate (Millipore, Durapore). For samples used in polony analyses of free cyanophages, the 0.2 µm filtrate was frozen as is at -80 ºC. For samples used in virus-like particle analyses, formaldehyde (2% final concentration) was added to the filtrate, incubated for 15-30 minutes in the dark, and stored at -80 ºC. Discrete samples used for validation of picocyanobacteria abundances determined by SeaFlow and for enumeration of heterotrophic bacteria abundances were collected and amended with glutaraldehyde (0.2% final concentration), incubated for 15-30 minutes in the dark, and snap frozen.

Data were recorded by Angelicque White, Michael Carlson, Francois Ribalet, Katie Watkins-Brandt, Nitzan Shamir, Julia Weissenbach, Ilia Maidanik, Sara Ferron, Bryndan Durham, Oscar Sosa, and Yotam Hulata. Data were recorded either automatically when sampled continuously in electronic files or in a sampling log.

Timing and spatial scale

Cruise tracks were designed to transit between the subtropical gyre to the subpolar gyre and cover the most ground in between given the ship time allotted. Each transect covered ~2000-2500 km. Underway flow cytometry and particulate carbon were sampled continuously. Discrete samples (for virus and cellular analyses and water chemistry) were taken every 4 or 8 hours over the sampling period. Bacterial uptake experiments were conducted so that experiments were performed at a spatial scale to evenly sample the environmental gradients on the 2017 cruise.

Dates of sampling and spatial scale of the transects were as follows:
March 21 – March 27, 2015, 3191.0 km transect
April 20 – May 1, 2016, 1591.0 km transect, 2 transect traversals
May 29 – June 10, 2017, 1839.5 km transect, 2 transect traversals
April 10 – April 27, 2019, 2188.7 km transect, 2 transect traversals

Data exclusions

No data were excluded from analyses.

Reproducibility	The 2016, 2017, and 2019 cruise were designed to traverse the same longitude line and were out-and-back transects.<br><br>All polony reactions were performed with at least technical duplicates. Select polony samples were repeated by two different people to assess potential systematic bias (none found). Additionally, select polony reactions for infected cells were run in separate labs, using different cytometers and reagents to verify data reproducibility.<br><br>Bacterial and viral abundances analyzed via flow cytometry or epifluorescent microscopy were run with 2-3 technical replicates.<br><br>Bacterial uptake experiments were preformed in triplicate.<br><br>All attempts at reproducibility were successful.
Randomization	Discrete measurements were analyzed in a random order. All samples were analyzed. For virus-like particle and polony measurements, fields of measurement were chosen at random. No experimental groups were needed in this analysis as no grouping existed.
Blinding	Blinding was performed to the extent that samples were given alphanumeric codes and thus analyzed without reference to collection site to minimize observer bias. No a priori expectations existed for these samples as they are the first to assess latitudinal gradients in cyanophage abundance and infection.

Did the study involve field work? ☒ Yes ☐ No

Field work, collection and transport

Field conditions	The upper mixed layer of the subtropical and subpolar gyres, and the transition zone between them in the North Pacific Ocean.<br>Environmental conditions ranged between:<br>Temperature: 10.8 – 26.3 °C<br>Salinity: 32.5 – 35.5<br>Nitrate+nitrate: <0.009 – 5.87 µM<br>Phosphate: 0.023- 0.513 µM<br>Particulate carbon: 0.67 – 18.3 µM<br>Mixed layer depth: 10-116 m
Location	Sampling was conducted between 5-15m depth in the North Pacific Ocean. The cruise tracks transited between:<br>(year: southernmost lat, long - northernmost lat, long)<br>2015: 24.55N, -153.12W - 44.05 N, 127.27 W<br>2016: 23.49 N, 157.98 W - 37.84 N, 158.00 W<br>2017: 25.77 N, 158.02 W - 42.35 N, 158.00 W<br>2019: 22.46 N, 158.00 W - 42.20 N, 157.6 W
Access & import/export	Sampling location were accessed on the RVs Kilo Moana, Ka'imikai O Kanaloa, and Marcus G. Langseth. All materials were imported and exported in compliance with local and international laws. No permits were needed.
Disturbance	No disturbance was caused by these cruises.

Reporting for specific materials, systems and methods

We require information from authors about some types of materials, experimental systems and methods used in many studies. Here, indicate whether each material, system or method listed is relevant to your study. If you are not sure if a list item applies to your research, read the appropriate section before selecting a response.

Materials & experimental systems

n/a	Involved in the study	
☒	☐	Antibodies
☒	☐	Eukaryotic cell lines
☒	☐	Palaeontology and archaeology
☒	☐	Animals and other organisms
☒	☐	Human research participants
☒	☐	Clinical data
☒	☐	Dual use research of concern

Methods

n/a	Involved in the study	
☒	☐	ChIP-seq
☐	☒	Flow cytometry
☒	☐	MRI-based neuroimaging

Flow Cytometry

Plots

Confirm that:

☒ The axis labels state the marker and fluorochrome used (e.g. CD4-FITC).

☒ The axis scales are clearly visible. Include numbers along axes only for bottom left plot of group (a 'group' is an analysis of identical markers).

☒ All plots are contour plots with outliers or pseudocolor plots.

☒ A numerical value for number of cells or percentage (with statistics) is provided.

Methodology

Sample preparation	For analyses of infection levels, water was first filtered through a 20 µm mesh. Ten milliliter samples were collected for iPolony infected cell analysis, amended with glutaraldehyde (0.1% final concentration), incubated at 4 ºC in the dark for 15-30 minutes, snap frozen in liquid nitrogen, and stored at -80 ºC. Discrete samples used for validation of picocyanobacteria abundances determined by SeaFlow and for enumeration of heterotrophic bacteria abundances were collected and amended with glutaraldehyde (0.2% final concentration), incubated for 15-30 minutes in the dark, and snap frozen.<br><br>Prior to running, samples were thawed at 30 degrees C in a water bath in the dark and transferred to ice once thawed. All samples were amended with 1 µm yellow-green beads as an internal reference. Heterotrophic bacteria were analyzed from discrete samples by staining each sample with SYBR Green (1X final concentration) for 15 minutes in the dark on ice.
Instrument	SeaFlow or a BD Influx flow cytometer
Software	Continuous flow cytometery measurements were collected using SeaFlow software (Swalwell et al. 2011)<br>Discrete flow cytometry data were acquired using FACSDiva v8 or Spigot software
Cell population abundance	Prochlorococcus and Synechococcus cells were typically sorted at concentrations between 800-1000 cells per ul. Sorted cell populations were >99% pure as determined by fluorescence signatures of sorted cells.
Gating strategy	Synechococcus was gated and sorted based on their orange autofluorescence (phycoerythrin containing cells) and size based on forward scatter. Prochlorococcus was gated and sorted based on red autofluorescence (chlorophyll containing cells) and size based on forward scatter. Hierarchical gating based on orange then red fluorescence was employed to discriminate between Prochlorococcus and Synechococcus from mixed communities. This gating strategy has been extensively documented in previous literature (e.g. Chisholm et al. Nature 1988, Swalwell et al. Limnol. Oceanogr. Meth. 2011, Casey et al. Deep Sea. Res. II 2013, Thyssen et al. Front. Microbiol. 2014, Rii et al. Mar. Ecol. Prog. Ser. 2016).

☒ Tick this box to confirm that a figure exemplifying the gating strategy is provided in the Supplementary Information.
